# Long non‐coding small nucleolar RNA host genes in digestive cancers

**DOI:** 10.1002/cam4.2622

**Published:** 2019-11-06

**Authors:** Huan Yang, Zheng Jiang, Shuang Wang, Yongbing Zhao, Xiaomei Song, Yufeng Xiao, Shiming Yang

**Affiliations:** ^1^ Department of Gastroenterology Xinqiao Hospital Army Medical University Chongqing China; ^2^ Department of Gastroenterology The First Affiliated Hospital of Chongqing Medical University Chongqing China; ^3^ Department of Gastroenterology People's Hospital of Changshou Chongqing Chongqing China

**Keywords:** cancer, digestive, LncRNA, SNHG

## Abstract

Although long noncoding RNAs (lncRNAs) do not have protein coding capacities, they are involved in the pathogenesis of many types of cancers, including hepatocellular carcinoma, cervical cancer, and gastric cancer. Notably, the roles of lncRNAs are vital in nearly every aspect of tumor biology. Long non‐coding small nucleolar RNA host genes (lnc‐SNHGs) are abnormally expressed in multiple cancers, including urologic neoplasms, respiratory tumors, and digestive cancers, and play vital roles in these cancers. These host genes could participate in tumorigenesis by regulating proliferation, migration, invasion and apoptosis of tumor cells. This review focuses on the overview of the roles that lnc‐SNHGs play in the formation and progression of digestive cancers.

## Graphical abstract

This review provides an overview of the roles of lncSNHGs (long non‐coding small nucleolar RNA host genes) in digestive cancers.

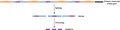

## 1 INTRODUCTION

Various studies have shown that the carcinogenic effects of genes are mainly exerted by transcription and protein encoding of genes.^1^, ^2^, ^3^ However, recent studies have shown that less than 2% of the human genome is coding genes, and over 90% of the genes are noncoding genes that play regulatory roles in most systems.^4^ Noncoding RNA (ncRNA) can regulate gene expression at different levels such as epigenetic modification, transcription and posttranscription.^5^, ^6^, ^7^ NcRNAs can be divided into short ncRNAs, midsize ncRNAs and lncRNAs according to the length of their nucleotides.^8^ Their lengths are 50, 50‐200, and more than 200 nucleotides, respectively.^9^ Long noncoding RNAs (lncRNAs) are longer than 200 nucleotides, which participate in the development of tumors in many ways.^10^, ^11^, ^12^ LncRNAs can directly or indirectly interact with target genes at the transcriptional level.^13^ At the same time, they can regulate histone modification and chromatin remodeling,^14^, ^15^ as well as affect other RNA generations.^16^ Additionally, they can act as competitive endogenous RNAs (ceRNAs) or precursors to small RNA molecules.^17^, ^18^

**Figure 1:**
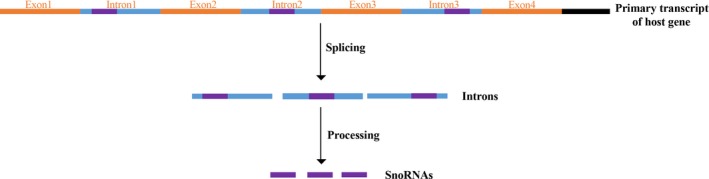
Primary RNA transcripts of host genes are spliced to many exons and introns. The removed introns that contain snoRNAs are processed further to mediated series of functions in the nucleolus

Small nucleolar RNA host genes (SNHGs) are host genes for snoRNAs. Primary RNA transcripts of host genes (including all exons and introns with their snoRNAs) are spliced to many exons and introns. Exons can play roles in the cytoplasm, and the removed introns that contain snoRNAs are processed further to mediated series of functions in the nucleolus (Figure 1). SnoRNAs consist of 60‐300 nucleotides and are mainly located in the nucleolus. They can be directly related to the posttranscriptional modification of some spliceosomal RNAs and ribosomal RNAs, and play crucial roles in the procession of useful ribosomes. Host genes include coding genes and noncoding genes. Long non‐coding small nucleolar host genes are one of the classes of SNHGs. Most snoRNAs are located in the introns of their host genes.^19^ Some scientists proposed that they might be regulated by host genes through cotranscription,^19^ but studies have also shown that the biological properties of host genes are independent of their snoRNA genes.^20^, ^21^

In the human genome, there are 232 host genes, including 15 non‐protein coding small nucleolar host genes,^22^ while recent studies have revealed increasing lnc‐SNHG members in cancers. Previously, researchers recognized that non‐coding snoRNA host genes contained only short, conservative, open reading frames without any known functions. However, recent studies have overturned this assumption. Long non‐coding small nucleolar host genes are found to be involved in the development of various diseases, including cancer progression, cell apoptosis and survival.^21^ Scientists have investigated many lnc‐SNHGs in multiple cancers. For instance, Lan et al described that the inhibition of *NUAK1* by *MIR‐145a‐5p* could inhibit the AKT pathway and reduce nasopharyngeal tumor cell invasion. However, *SNHG1* impaired the capacity of *MIR‐145a‐5p* to increase *NUAK1* and promoted nasopharyngeal carcinoma distant metastasis.^23^ Wang et al also discovered that *SNHG1* could inhibit *MIR‐302/372/373/520*’s influence on TGFB1/SMAD3 and RAB11A/Wnt signaling pathway to promote pituitary tumor cell growth, migration and metastasis.^24^ Researches have also shown that most lnc‐SNHG members play vital roles in digestive cancer progression. Li et al proposed that *SNHG5* could upregulate CTNNB1, MYC and CCND1 expression to activate the Wnt signaling pathway and then induce Epithelial‐mesenchymal transition (EMT) to promote liver cancer cell invasion.^25^ *SNHG17* bound with EZH2 and inhibited the expression of *CDKN2B* and *CDKN1C* to promote gastric cancer cell cycle progression.^26^ However, since the biofunctions, molecular mechanisms and potential pathways of SNHGs in digestive cancers are complicated, and they have not yet been clearly defined. Thus, we try to review them here for a better clarification.

## 2 LNC‐SNHGS IN DIGESTIVE CANCERS

In 1997, Mark et al first reported the small nucleolar RNA host gene *SNHG1* as the host gene of *SNORD22*. They detected the location of *SNHG1* in chromosome 11q13 and *SNORD22* in the nucleolus. There was little protein coding ability for the host gene of *SNORD22*.^27^ Subsequently, *SNHG3*,^28^ *GAS5*,^29^ *SNHG5* 30 were reported in succession, and hundreds of the genes have been researched to date. The Lnc‐SNHG family includes many members, in which the most associated with digestive cancers are *SNHG1*, *GAS (SNHG2)*, *SNHG3*, *SNHG5*, *SNHG6*, *SNHG7*, *SNHG8*, *SNHG9*, *SNHG12*, *DANCR (SNHG13)*, *SNHG14*, *SNHG15*, *SNHG16*, *SNHG17*, *SNHG18* and *SNHG20*. Multiple molecular regulatory mechanisms of each SNHG member are involved in different human cancers. Some SNHGs can act as sponges of microRNAs to inhibit the roles of microRNAs in tumorigenesis and affect tumor progression.^23^, ^31^, ^32^ On the other hand, they can also bind proteins to influence target genes or impact tumorigenesis via different signaling pathways, including the EMT, Wnt, PIK3CA, NF‐κB, and TP53 signaling pathways.^33^, ^34^, ^35^ Moreover, the relationship with transcriptional activation also participates in the progression.^36^ The biological effects are mainly exerted through the above mechanisms. However, additional studies are needed to learn more about the regulation and control of SNHG members. Here, according to the reported results, we have constructed a table describing each SNHG member's alternative name, relative snoRNAs, chromosome location, subcellular location, related pathways and associated digestive cancers types (Table 1).

**Table 1:** SNHG members in digestive cancers

SNHG member	Aliases	SnoRNAs	Chromosomal location	Main subcellular location	Related digestive cancer types	Interactions and related genes	Related pathway	Related cell bio‐functions	Role	References
*SNHG1*	*LINC00057, NCRNA00057, U22HG, UHG, lncRNA16*	*SNORD22, SNORD25, SNORD26, SNORD27, SNORD28, SNORD29, SNORD30, SNORD31*	11q12.3	Cytoplasm	Colon cancer; gastric cancer; liver cancer; esophageal cancer; cholangiocarcinoma；pancreatic cancer	*MIR497/MIR‐195‐5p, MIR145*, EZH2, *KLF2, CDKN2B*, HNRNPC, TP53, MYC, CTNNB1, MMP9, *MIR140, ADAM10, DNMT1, TP53, BAX, FAS, CDKNIA,* DNMT1, *MIR195, MIR338*, CST3, CASP8/3	EMT; TP53 pathway; AKT signaling pathway; Wnt signaling pathway; NOTCH signaling pathway	Proliferation, cycle, apoptosis, migration, invasion	Oncogene	[6, 7, 23, 24, 33, 38, 39, 40, 41, 42, 64, 65, 83, 84, 85, 86, 102, 112, 120]
*GAS5*	*SNHG2, NCRNA00030,*	*SNORD74, SNORD77, SNORD75, SNORD76, SNORD44, SNORD78, SNORD79, SNORD80, SNORD47, SNORD81*	1q25.1	Cytoplasm	Colon cancer; gastric cancer; liver cancer; esophageal cancer; pancreatic cancer	*MIR‐182‐5p, MIR‐221, FOXO3a, MIR222, MIR23A, MT2A,* YBX1, CDKN1A, *MIR21,* CDH1, VIM, *MIR301A*, MIR‐181c‐5p, MIR‐32‐5p, PTEN	PTEN/AKT/MTOR pathway; EMT; Wnt signaling pathway; NF‐kB pathway	Proliferation, cell cycle, apoptosis, migration, invasion	Antioncogene/oncogene	[29, 34, 36, 43, 44, 45, 46, 66, 67, 87, 88, 89, 90, 103, 104, 105, 113, 114]
*SNHG3*	*U17HG; RNU17C; RNU17D; U17HG‐A; U17HG‐AB; NCRNA00014*	*SNORA73A, SNORA73B*	1p35.3	Cytoplasm	Colon cancer; liver cancer	*CCNB1, CCND2, CDK4, E2F1, MIR‐182‐5p, MYC, MIR128, CD151*	EMT	Invasion, proliferation	Oncogene	[47, 91, 92]
*SNHG5*	*C6orf160, LINC00044, NCRNA00044, U50HG*	*SNORD50A, SNORD50B*	6q14.3	Cytoplasm	Colon cancer; gastric cancer; liver cancer;	*MIR‐132‐3p, CREB5, SPATS2,* STAU1, METase, *MIR20,* BECN1, ATG5, ATG7, LC3‐II/LC3‐I, *MIR32,KLF4,*MTA2,MMP2, MMP9, EGFR, CDH1, CDKN1A, *MIR‐26a‐5p, GSK3B*,CTNNB1, MYC, CCND1	Wnt signaling pathway; EMT	Apoptosis, proliferation, migration	Antioncogene/oncogene	[25, 31, 48, 49, 68, 69]
*SNHG6*	*HBII‐276HG, NCRNA00058, U87HG*	*SNORD87*	8q13.1;	Cytoplasm	Colon cancer; gastric cancer; liver cancer; esophageal cancer	EZH2*, CDKN1A, MIR760, FOXC1, MIR‐101‐3p, ZEB1, CDH2*, EZH2, *CDKN1B*, MAPK1, MAPK8, MAPK14, TP53, EZH2, *CDKN1A*, *MIR‐101‐3p,* UPF1	EMT; JNK signaling pathway; TGFB1/SMAD signaling pathway;	Proliferation, apoptosis, migration, invasion	Oncogene	[50, 51, 52, 70, 71, 107, 108]
*SNHG7*	*NCRNA00061*	*SNORA17A, SNORA17B*	9q34.3	Cytoplasm	Colon cancer; gastric cancer; liver cancer; esophageal cancer;	*B3GLCT, FUT2, MFNG, MGAT4A, GALNT1, GALNT5, GALNT7, ST3GAL5, ST6GALNAC2, MIR216B,* CDKN2B, CDKN2A	PIK3CA/AKT/MTOR signaling pathway; EMT	Proliferation, migration, invasion, apoptosis, cell cycle progression	Oncogene	[32, 53, 72, 93, 106]
*SNHG8*	*LINC00060, NCRNA00060*	*SNORA24*	4q26	Cytoplasm	Liver cancer	*MIR149*	—	Proliferation, cell cycle, apoptosis, migration, invasion	Oncogene	[94]
*SNHG9*	*NCRNA00062*	*SNORA78*	16p13.3	—	Pancreatic cancer	—	—	Proliferation	Antioncogene	[115]
*SNHG12*	*C1orf79; PNAS‐123; LINC00100; ASLNC04080; NCRNA00100*	*SNORA44，SNORA61，SNORA16A, SNORD99*	1p35.3	Cytoplasm	Colon cancer; gastric cancer; liver cancer;	CDK4, CDK6, CCND1, CASP3, *MIR‐199a/b‐5p, MIR320, MIR‐199a/b‐5p*	NF‐κB signaling pathway	Proliferation, cell cycle, apoptosis	Oncogene	[54, 73, 74, 95]
*DANCR*	*AGU2; ANCR; SNHG13; KIAA0114; lncRNA‐ANCR*	*SNORA26*	4q12	*—*	Colon cancer; gastric cancer; liver cancer	*MIR577, HSPB1, NPTN‐IT1,* EZH2, HDAC3, CTNNB1	—	Proliferation, invasion, migration	Oncogene	[55, 56, 75, 76, 96]
*SNHG14*	*115HG, IC‐SNURF‐SNRPN, LNCAT, NCRNA00214, U‐UBE3A‐ATS, UBE3A‐AS, UBE3A‐AS1, UBE3A‐ATS, UBE3AATS*	*SNORD115‐(1 ~ 48), SNORD116‐(1 ~ 30), SNORD (64,107,108)*	15q11.2	Cytoplasm	Gastric cancer	*MIR‐145, SOX9*	PIK3CA/AKT/MTOR signaling pathway	Migration, invasion, apoptosis	Oncogene	[77]
*SNHG15*	*C7orf40, MYO1GUT, Linc‐Myo1g*	*SNORA9*	7p13	Nucleus	Colon cancer; gastric cancer; pancreatic cancer	SNAI2, MMP2, MMP9, EZH2, *CDKN2B, KLF2*	—	Proliferation, invasion, growth, migration, apoptosis	Oncogene	[57, 58, 78, 116, 117]
*SNHG16*	*Nbla10727, Nbla12061, ncRAN*	*SNORD1A, SNORD1B, SNORD1C*	17q25.1	Cytoplasm	Colon cancer; gastric cancer; esophageal cancer; liver cancer	*MIR‐140‐5p,* MYC, CTNNB1, CCND1, *ZEB1*	Wnt signaling pathway	Proliferation, migration, invasion, cell cycle, apoptosis	Antioncogene/oncogene	[59, 97, 109, 110]
*SNHG17*	*9430008C03Rik*	*SNORA71A, SNORA71B, SNORA71C, SNORA71D*	20q11.23	Nucleus	Colon cancer; gastric cancer	EZH2, *CDKN1C, CDKN2B*	*—*	Proliferation, cell cycle	Oncogene	[26, 60]
*SNHG18*	*—*	*SNORD123*	5p15.31	*—*	Liver cancer	*—*	*—*	*—*	*—*	*—*
*SNHG20*	*C17orf86, LINC00338, NCRNA00338, SCARNA16HG*	*SCARNA16*	17q25.2	Cytoplasm	Colon cancer; gastric cancer; liver cancer	*MIR‐495‐3p, ZFX*	EMT	Proliferation, invasion, cell cycle	Oncogene	[35, 61, 79, 98]

## 3 BIOLOGICAL FUNCTIONS AND MECHANISMS OF SNHGs IN DIGESTIVE CANCERS

### 3.1 SNHGs in colorectal cancer

Among the causes of death from malignant tumors, colon cancer ranks the fourth in China.^37^ Although surgical resection is radical, the recurrence rate is still high. Moreover, some patients lost the chances of surgery when they are diagnosed. It is increasingly important to study the treatment of related genes in colon cancer. Higher *SNHG1* expression indicated a poor prognosis in colon cancer. *SNHG1* was determined to be an independent indicator for the poor prognosis of colorectal cancer.^38^, ^39^ *SNHG1* is located in both cytoplasm and nucleus. But different researchers have different conclusions about whether *SNHG1* mainly exists in the nucleus or cytoplasm. Bai J et al and Tian T et al showed that *SNHG1* was mainly located in the cytoplasm.^40^, ^41^ *SNHG1* could sponge *MIR‐497/MIR‐195‐5p* to influence EMT to facilitate cancer cell migration and invasion,^40^ and sponge *MIR*‐*145* to increase the expression of *MIR‐145*’s targets, to promote colorectal cancer cell proliferation.^41^ On the contrary, Xu et al confirmed the presence of *SNHG1* mainly in the nucleus by performing in situ hybridization and nuclear slurry separation experiments. In the nucleus, *SNHG1* combined with protein EZH2 to decrease the epigenetic impact of *KLF2* and *CDKN2B*.^42^ Additionally, downregulation of *SNHG1* could promote apoptosis and reduce the size and weight of tumors in vivo.^38^ Yuan Shen et al^33^ also found that *SNHG1* could undergo nuclear residency on activation of doxorubicin, and nuclear‐resident *SNHG1* competitively binds HNRNPC to weaken the association between HNRNPC and TP53, and to increase TP53’s expression level, transcriptional activity and phosphorylation, leading to TP53‐dependent apoptosis of colon cancer cells.^33^ *SNHG1* also accelerated colorectal cancer tumorigenesis by affecting MYC, CTNNB1, MMP9, and activated the Wnt signaling pathway.^33^

*SNHG2*, known as *GAS5*, is a tumor suppressor gene that has been thoroughly studied previously. Downregulation of *GAS5* was significantly connected with high malignant level and lymphatic metastasis.^43^ Reduced *GAS5* facilitated colon cancer cell migration, proliferation and invasion.^44^ Downregulated *GAS5* also promoted the cell cycle in the G0/G1 stage and prevented apoptosis. Increased *GAS5* was an independent marker of a longer overall survival and better prognosis.^43^, ^45^Yuan et al found that co‐overexpressed *GAS5* and *SNORD44* could significantly repress tumor growth and they could induce cancer cells apoptosis in both vivo and vitro.^46^ The overexpression of *GAS5* could suppress colon cancer cells proliferation and promote apoptosis by inhibiting the expression of *MIR‐182‐5p* and *MIR‐221*, but upregulating *FOXO3a*.^43^, ^44^

*SNHG3* acted as an oncogene and accelerated cancer cell growth in tumorigenesis.^47^ In colorectal cancer, *SNHG3* could augment the expression of *MYC* and *MYC*’s target genes *CCNB1*, *CCND2*, *CDK4* and *E2F1*. Combining the results of GSE54632 database and starBase2.1, Huang et al found that *MIR‐182‐5p* was the only gene that could target *MYC* and at the same time, be sponged by *SNHG3*. *SNHG3* obstructed *MIR‐182‐5p's* suppression on *MYC* to promote tumor growth.^47^

*SNHG5* sponged *MIR‐132‐3p* and positively regulated *CREB5* to inhibit colon cancer cell apoptosis but promoted cancer cell proliferation, migration and invasion.^48^ The upregulation of *SNHG5* led to the increased mRNA expression of *SPATS2*, and *SNHG5* played this role by decreasing the effect of the protein STAU1 on *SPATS2* to promote colon cancer proliferation.^49^ Li and Li et al verified that the expression of *SNHG6* in colon cancer tissues was higher than that in normal tissues.^50^, ^51^ *SNHG6* could induce EZH2 to bind with the promoter of *CDKN1A* and inhibit the *CDKN1A* function, leading to the growth of cancer cells.^51^ In the cytoplasm, *SNHG6* sponged *MIR760* and upregulated its target gene *FOXC1* to promote colon cancer cell proliferation, migration and invasion.^52^ *SNHG7* was located in the cell cytoplasm in colon cancer and was highly expressed in colon cancer tissues.^32^ Although several mRNAs, including *B3GLCT*, *FUT2*, *MFNG*, *MGAT4A*, *GALNT1*, *GALNT5*, *GALNT7*, *ST3GAL5*, and *ST6GALNAC2* were related, *GALNT7* was the most commonly associated with *SNHG7*. The overexpression of *SNHG7* and *GALNT1* could enhance cell proliferation and invasion. Additionally, *GALNT1* was the direct target gene of *MIR216B*, and *SNHG7* could act as a ceRNA to sponge *MIR216B*, and rescue *GALNT1* to facilitate colon cancer cell invasion. Li Y et al further pointed out that the PIK3CA/AKT/MTOR signaling pathway might play a crucial role in the mechanism induced by *SNHG7*.^53^ *SNHG7* also could sponge *MIR216B*, to increase *GALNT1* and activate EMT to promote colorectal cancer cell migration and invasion.^32^ Wang et al put forward that *SNHG12* augmented colon cancer proliferation, cell cycle progression and inhibited apoptosis by inhibiting the related proteins CDK4, CDK6 and CCND1, and suppressing CASP3.^54^ *S*horter overall survival rate and disease‐free survival rate were correlated with higher *DANCR* expression. It was determined as an individual poor prognosis factor in colon cancer.^55^, ^56^ DANCR could upregulate colon cancer cell proliferation and migration ability by sponging *MIR577* and increase the expression of *HSPB1*.^56^ Huang et al and Zhang et al concluded that *SNHG15* and *SNHG16* were both highly expressed in colon cancer samples.^57^, ^58^, ^59^ *SNHG15* could only increase the protein level of SNAI2 but not the mRNA level through preventing SNAI2’s ubiquitin.^58^ Wnt signaling pathway and ceRNA mechanism might participate in the progression of *SNHG16*.^59^ *SNHG17* could bind with EZH2 and regulate *CDKN1C* to promote cell proliferation.^60^ *SNHG20* promoted cancer cell proliferation, migration and invasion, but the flow cytometry results showed that *SNHG20* was only related to cell cycle progression and had no relationship with cell apoptosis.^61^

### 3.2 SNHGs in gastric cancer

Gastric cancer is the third leading cause of death worldwide.^62^, ^63^ As described previously in colon cancer tissues, the patients also showed the short life time when the expression of *SNHG1* was significantly high. Knocking down the expression of *SNHG1* could reduce the tumor size and suppress cell proliferation and colony formation. Similarly, *SNHG1* also sponged miRNA in the cytoplasm. *SNHG1* inhibited the expression of *MIR140* and upregulated the expression of *ADAM10* to increase the ability of proliferation and invasion of gastric cancer cells.^64^ Additionally, *SNHG1* could also promote the proliferation of gastric cancer cells by upregulating the expression of *DNMT1*.^65^

*GAS5*, a tumor inhibitor gene, inhibits gastric cancer cell proliferation, blocks the cell cycle and promotes cell apoptosis.^34^, ^36^, ^66^ Li Y et al and Liu X et al showed that *GAS5* sponged *MIR222* and *MIR23A* in gastric cancer tumorigenesis.^34^, ^67^ Li et al further studied that *GAS5* could bind *MIR222* and regulate the PTEN/AKT/MTOR pathway to decrease gastric cancer proliferation.^34^ Another study verified that *GAS5* combined with the 3'UTR of *MIR23A* and inhibited the effect of *MT2A* to impair gastric cancer progression.^67^ Additionally, the downregulation of *GAS5* could only obstruct the protein level of transcriptional activator Y‐box binding protein 1 (YBX1), but not reduce its mRNA level. Downregulated *GAS5* interacted with YBX1 to reduce the expression of CDKN1A and promote the cell cycle.^36^ In accordance with *GAS5*, *SNHG5* could also facilitate gastric cancer cell apoptosis.^68^ Moreover, it could reduce cancer cell proliferation and migration.^31^ The subcellular location of *SNHG5* was mainly in the cytoplasm.^69^ *SNHG5* was found to be the target gene of L‐methionine‐α‐deamino‐γ‐mercaptomethane‐lyase (METase). Increased METase promoted gastric cancer cell apoptosis by upregulating the expression of *SNHG5*. Upregulated *SNHG5* reduced *MIR20A* and led to the overexpression of the apoptosis proteins BECN1, ATG5, ATG7 resulting in increased proportion of LC3‐II/LC3‐I.^68^ Additionally, *SNHG5* could sponge *MIR32,* and* MIR32* could reduce the migration and proliferation effects of *SNHG5* on gastric cancer cells. Conversely, when *MIR32* inhibited its target gene *KLF4*, the overexpression of *SNHG5* could partially prevent *MIR32* function.^31^ Moreover, Zhao et al found that upregulation of *SNHG5* could prevent MTA2 locating to the nucleus from the cytoplasm, and inhibit gastric cancer cell migration and invasion.^69^ They also found that when *SNHG5* was overexpressed, the protein levels of MMP2, MMP9 and EGFR were reduced, while CDH1 and CDKN1A were upregulated.^69^

In gastric cancer, *SNHG6* was significantly highly expressed in gastric cancer tissues and in serum.^70^, ^71^ Yan K et al suggested that high expression of *SNHG6* was related to the tumor grade and lymph node metastasis, which predicted a poor prognosis for patients.^70^ Yan et al and Li et al both proposed that *SNHG6* existed not only in the cytoplasm but also in the nucleus, and the proportion in the cytoplasm was nearly 67.5%‐80%. It participated in both transcriptional and posttranscriptional regulation.^70^, ^71^ In the cytoplasm, *SNHG6* could suppress *MIR‐101‐3p*, upregulate *ZEB1* and *CDH2*, and accelerate EMT progression.^70^ In the nucleus, *SNHG6* could recruit EZH2 to the promoter of *CDKN1B* to play the transcriptional regulatory role.^70^ In another study, downregulation of *SNHG6* could augment the phosphorylation level of MAPK1, MAPK8 and MAPK14, while increasing the expression of TP53 and decreasing the expression of EZH2. Reduced *SNHG6* enhanced the expression of *CDKN1A* via the JNK signaling pathway to participate in tumor growth.^71^ Down‐regulated *SNHG7* could arrest gastric cancer cell cycle progression in the G0‐G1 period probably because it augmented the expression of CDKN2B and CDKN2A.^72^ Yang et al and Zhang et al raised the idea that not only *SNHG12* accelerated gastric cancer cell proliferation and invasion, but it also determined the adverse events prediction.^73^, ^74^ *SNHG12* could sponge *MIR‐199a/b‐5p* and *MIR320* to promote tumorigenesis.^73^, ^74^ *DANCR* and *SNHG14* were also upregulated in gastric cancer and could promote cancer cell proliferation, invasion and migration.^75^, ^76^, ^77^ Mao et al described that *DANCR* could also regulate another lncRNA. They found *DANCR* inhibited the expression of *NPTN‐IT1* by binding with EZH2 and HDAC3.^76^ *SNHG14* suppressed the inhibition of *MIR‐145* on *SOX9*, and activated the PIK3CA/AKT/MTOR signaling pathway to accelerate cell proliferation and invasion.^77^ *SNHG15* was expressed at a higher level in cancer tissues, with expression increased by over 1.5‐fold compared to the normal tissues. It promoted cell invasion and migration by increasing MMP2 and MMP9.^78^ *SNHG17* bound with EZH2 and inhibited the expression of *CDKN2B* and *CDKN1C* to promote gastric cancer cell cycle progression in the G0/G1 phase.^26^ Liu et al further proposed that *SNHG20* could facilitate gastric cancer cell proliferation and invasion.^35^ Another study showed *SNHG20* was located in the cytoplasm, and *SNHG20* interacted with *MIR‐495‐3p* to upregulate *ZFX*, and promoted gastric tumor growth and invasion.^79^

### 3.3 SNHGs in liver cancer

The morbidity and mortality of liver cancer are still high in the world.^80^, ^81^ α‐fetoprotein (AFP) was a crucial factor in predicting the occurrence and recurrence; however, Gao et al found that the high expression of *SNHG1* in the blood plasm was superior to AFP to distinguish liver cancer from the control group, and the combination of *SNHG1* and AFP could further improve the ability of distinguishing hepatic cancer.^82^ Gao et al pointed out that upregulated *SNHG1* was associated with advanced tumor, TNM stage and AFP level, but did not correlate with age and smoking status.^82^ *SNHG1* promoted liver cancer cell cycle and inhibited apoptosis by suppressing the expression of *TP53*’s target genes, including *BAX*, *FAS*, and *CDKNIA*.^83^ Li et al further found that *SNHG1* reduced *TP53* by binding the protein DNMT1, and the overexpression of *TP53* could partially impair the effect of *SNHG1* on cancer tumorigenesis.^84^ Other scientists researched the impact of *SNHG1* on sorafenib resistance.^85^ Overexpression of *SNHG1* could significantly enhance the sorafenib resistance of liver cancer.^85^ Moreover, *SNHG1* sponged *MIR195* to promote cancer cell proliferation and metastasis.^86^

On the contrary, reduced expression of *GAS5* was associated with poor differentiation, advanced TNM stage, tumor size, lymph node metastasis and acted an independent poor prognosis marker for liver cancer.^87^, ^88^ *GAS5* could downregulate *MIR21* to prevent cancer cell migration and invasion.^89^ Chang et al indicated that *GAS5* repressed cell proliferation by means of reducing VIM, increasing CDH1 and influencing EMT pathway.^90^

The overexpression of *SNHG3* predicted high rates of larger tumor size, portal vein tumor thrombus, sorafenib resistance and relapse.^91^, ^92^ It directly combined with *MIR128* to upregulate the expression *CD151*, and activated EMT to promote cell invasion.^91^ Li found that the overexpression of *SNHG5* inhibited the suppressive influence of *MIR‐26a‐5p* on *GSK3B* to promote liver cancer tumorigenesis. Additionally, when *SNHG5* increased *GSK3B* expression, CTNNB1, MYC, and CCND1 were upregulated to activate the Wnt signaling pathway and then induced EMT to promote cancer cell invasion.^25^ Cui et al analyzed several datasets from TCGA and GEO database. They found two significantly differentially expressed lncRNAs, named *PVT1* and *SNHG7*. Cell biofunction experiments verified that *SNHG7* could increase cell invasion ability,^93^ which implied that *SNHG7* acted as an oncogene to promote tumorigenesis. Dong et al regarded that *SNHG8* promoted liver cancer tumorigenesis and pulmonary metastasis via sponging *MIR149*.^94^ *SNHG12* was the host gene of four small nucleolar RNAs—*SNORA44*, *SNORA61*, *SNORA16A* and *SNORD99*. *SNHG12* was expressed at a significantly higher level in cancerous tissues than in normal tissues. But the change of the expression of *SNHG12* did not cause expression fluctuation in the four small nucleolar RNAs.^95^ *SNHG12* located mainly in the cytoplasm. Its high expression was related to tumor size, TNM stage, vascular invasion, and relapse and predicted a poor prognosis but was not involved in the AFP level, portal vein tumor thrombosis, tumor differentiation, gender and age. *SNHG12* promoted liver cancer cell proliferation and invasion, and resulted in a marked reduction in apoptosis.^95^ *SNHG12* also sponged *MIR‐199a/b‐5p*, which directly targeted the key markers of the NF‐κB signaling pathway.^95^ Similar to *SNHG1*, the high expression level of *DANCR* might be a more advanced marker than AFP to identify hepatic cancer no matter in the sensitivity or specificity. *DANCR* might promote cancer cell proliferation and invasion by inhibiting protein CTNNB1.^96^ On the contrary, Xu et al showed that *SNHG16* acted as an antioncogene in hepatocellular carcinoma. *SNHG16* was expressed at a lower level in the cancerous tissue than normal tissue, and *SNHG16* could alleviate 5‐FU resistance.^97^ Liu J et al showed that *SNHG20* played an oncogenic role in liver cancer, and promoted the EMT pathway in cancer progression.^98^

### 3.4 SNHGs in esophageal cancer

Esophageal cancer is a common digestive system tumor^99^ with the number of cases increasing annually; more than 300,000 people die from this cancer each year.^100^ In 2016, the numbers of new cases and fatal cases of esophageal cancer in the United States were approximately 16 910 and 15 910, respectively,^101^ indicating the increased morbidity and mortality of esophageal cancer. The discovery of long noncoding RNAs provides further clinical idea for the diagnosis and treatment of esophageal cancer. *SNHG1* was significantly upregulated in esophageal cancer tissues. It also promoted the proliferation, cloning, and invasion of esophageal cancer cells.^6^, ^102^ *SNHG1* could activate the NOTCH and EMT pathway to augment cancer cell invasion and growth, while *SNHG1* sponged *MIR338* to increase the expression of CST3 and to downregulate CASP8/3.^6^, ^102^ Regarding *GAS5*, in contrast to other digestive cancers types, Li W et al showed that *GAS5* no longer acted as a tumor suppressor gene but acted as an oncogene in esophageal cancer. It could sponge *MIR301A* to affect Wnt and NF‐kB signaling pathways to promote cancer cell proliferation, migration and invasion but reduced cell apoptosis.^103^ However, Ke et al insisted that *GAS5* was an anticancer gene in esophageal tumor. Overexpression of *GAS5* significantly impeded tumorigenesis via EMT.^104^ Huang et al verified the influences of *GAS5* on proliferation, invasion and migration, which was consistent with Ke K's opinions.^105^ *SNHG6* and *SNHG7* were both expressed higher in esophageal cancer tissues than normal tissues, and promoted cancer cell proliferation and metastasis.^106^, ^107^, ^108^ Xu et al suggested that CDKN2B and CDKN2A were partially connected to *SNHG7* in the proliferation and metastasis progression.^106^ Additionally, reduced *SNHG16* resulted in the downregulation of key markers of the Wnt signaling pathway, such as MYC, CTNNB1, and CCND1.^109^ Furthermore, *SNHG16* showed a positive correlation with *ZEB1* to promote esophageal cancer tumorigenesis by sponging MIR‐140‐5p.^110^

### 3.5 SNHGs in pancreatic cancer

The incidence of pancreatic cancer ranks the eleventh worldwide. The incidence and mortality of pancreatic cancer in developed countries are higher than those in developing countries. In 2012, approximately 338 000 people had pancreatic cancer, and the number of deaths exceeded 331 000. Li et al found that *SNHG1* not only promoted pancreatic cancer tumorigenesis, but also was differently expressed in gemcitabine‐resistant and gemcitabine‐sensitive pancreatic cells, which suggested that *SNHG1* could play an important role in tumor therapy. The phosphatidylinositol 3‐kinase‐AKT signaling pathway might affect this drug resistance.^111^ Cui et al suggested that *SNHG1* could upregulate the key markers of the NOTCH signaling pathway to affect pancreatic cancer proliferation and invasion.^112^ Additionally, *SNHG1* also played a crucial role in pancreatic ductal adenocarcinoma. The PIK3CA/AKT signaling pathway was activated when *SNHG1* was overexpressed.^39^ Gao et al put forward that *GAS5* reduced the drug resistance of cancer cells through regulating *MIR‐181c‐5p* and Hippo pathway.^113^ Moreover, *GAS5* could downregulate *MIR‐32‐5p* and increase the PTEN protein level.^114^ *SNHG9* was expressed at a lower level in cancer tissues and serum than in normal tissues, and there were negative correlations with cancer stage, lymph node metastasis, disease prognosis. *SNHG9* played an antioncogenic role and decreased pancreatic cancer cell proliferation.^115^ *SNHG15* was mainly located in the nucleus; high expression of *SNHG15* predicted a poor differentiation of pancreatic cancer. In the nucleus, *SNHG15* could bind with EZH2 to the promoter of *CDKN2B* and *KLF2* to inhibit their expression.^116^, ^117^

### 3.6 SNHGs in other digestive cancer types

Cholangiocellular carcinoma is a type of tumor with high invasive character.^118^ The survival time of most patients is only 2 years after the diagnosis.^119^ Yang et al^120^ researched The Cancer Genome Atlas CCA, RNA Sequencing data and Gene Expression Omnibus GSE76297 and concluded that *SNHG1* was expressed at a higher level in cholangiocarcinoma tissues than in normal tissues. Upregulated *SNHG1* could promote cholangiocarcinoma cell proliferation, migration, and cell cycle but reduce apoptosis,^121^, ^122^ and the interaction between *SNHG1* and EZH2 could target *CDKN1A* to promote the biological behavior of cholangiocarcinoma.^120^

## 4 SMALL NUCLEOLAR HOST GENES AND snoRNAs

SnoRNAs could be regulated by their host genes, copy number variation, and DNA methylation.^19^ Some scientists pointed out that the host genes may affect the expression of snoRNAs by cotranscription^19^; however, other scholars reported that the functions of some SNHG members were independent of their snoRNAs.^21^ Moreover, recent studies have shown that some snoRNAs are also related to cancer tumorigenesis.^21^ Scholars have reported that some snoRNAs can produce smaller products during nucleolytic processing, and these products, like microRNAs, can play important roles in tumor progression.^123^ Some researchers call these products as sno‐miRNAs.^124^ Long noncoding RNAs can sponge microRNAs in the cytoplasm, but it is still unclear whether there is a potential pathway by which lnc‐SNHGs and snoRNAs jointly regulate microRNAs, which is worthy of further exploration.

## 5 CONCLUSION

It has been shown that the irregular expression status of SNHGs is significantly related to digestive tumors stage, metastasis, infiltration, and poor prognosis in cancers. SNHGs also act as prognostic factors in most malignant tumors. Many studies have implied that SNHG members regulate the development of tumor diseases by the means of mediating its sponge miRNAs, activating different signaling pathways, and regulating the expression of key markers. However, these studies are just preliminary discussions; further mechanistic studies on SNHG members and snoRNAs will be required in the future.

## CONFLICT OF INTEREST

The authors declare no conflict of interest.

## AUTHOR CONTRIBUTIONS

S‐M Y, Y‐F X and HY designed the study and drafted the manuscript. HY wrote the paper. ZJ revised the paper. X‐M S, SW and Y‐B Z received and reviewed the manuscript. All authors read and approved the manuscript and agree to be accountable for all aspects of the research.
